# Omega Fatty Acid and Protein Profiles of Colostrum and Transitional Milk in Mexican Women With and Without Gestational Diabetes: A Cross-Sectional Study

**DOI:** 10.3390/nu18111803

**Published:** 2026-06-03

**Authors:** Larissa Martínez-Ortega, Carlos A. Ibáñez, Isabel Omaña-Guzmán, Consuelo Lomas Soria, José Leopoldo Aguilar Faisal, Omar Granados Portillo, Ana Méndez Carballo, Emilia Lozano González, Fausto Coronel Cruz, José Carranco Martínez, Víctor Carmona Ornelas, Nayely Garibay-Nieto, Elena Zambrano

**Affiliations:** 1 Department of Reproductive Biology, Instituto Nacional de Ciencias Médicas y Nutrición “Salvador Zubirán” (INCMNSZ), Mexico City 14080, Mexico; larimartinez98@gmail.com (L.M.-O.); carlos_albertoibc@hotmail.com (C.A.I.); consuelo.lomass@incmnsz.mx (C.L.S.); 2 Child Wellness Unit, Department of Pediatrics, Hospital General de México Dr. Eduardo Liceaga, Mexico City 06720, Mexico; nutricion.isa@gmail.com (I.O.-G.); anamendezcarballo@gmail.com (A.M.C.); emilia.lozano.el@gmail.com (E.L.G.); 3 Department of Biology, Facultad de Química, Universidad Nacional Autónoma de México, Mexico City 04510, Mexico; 4 Laboratory of Conservation Medicine, Escuela Superior de Medicina, Instituto Politécnico Nacional, Mexico City 11340, Mexico; jaguilarf@ipn.mx; 5 Department of Nutritional Physiology, Instituto Nacional de Ciencias Médicas y Nutrición “Salvador Zubirán” (INCMNSZ), Mexico City 14080, Mexico; omar.granadosp@incmnsz.mx; 6 Obstetrics and Gynecology Department, Hospital General de México Dr. Eduardo Liceaga, Mexico City 06720, Mexico; coronel1019@hotmail.com (F.C.C.); dr.albertocarranco@gmail.com (J.C.M.); victorh.carmona@gmail.com (V.C.O.)

**Keywords:** breastfeeding, colostrum, human milk composition, Polyunsaturated fatty acids, proteins

## Abstract

**Background/Objectives**: Gestational Diabetes Mellitus (GDM) involves metabolic alterations that may affect breast milk composition. Imbalances in protein and fatty acid (FA) profiles have been reported in mature milk from mothers with GDM. However, evidence for colostrum and transitional milk is limited, despite the key role of ω-3 and ω-6 Polyunsaturated fatty acids (PUFAs) in neonatal neurodevelopment. This study compared ω-3 and ω-6 PUFAs and protein concentrations in colostrum and transitional milk from women with and without GDM. **Methods**: This cross-sectional study was conducted from January 2023 to December 2024. Women aged ≥ 18 years with GDM and non-GDM pregnancies recruited at Hospital General de México “Dr. Eduardo Liceaga” were included. Colostrum and transitional milk samples were collected at 0–5 and 6–14 days postpartum, respectively. To assess whether postpartum time (hours) and maternal group (non-GDM vs. GDM) affected milk volume, an analysis of covariance (ANCOVA) was performed. Differences in milk composition between the GDM and non-GDM groups were assessed using Student’s *t* test or the Mann–Whitney U test, according to variable distribution. **Results**: A total of 71 milk samples were analyzed: 51 colostrum samples (25 from women with GDM and 26 from women with non-GDM) and 20 transitional milk samples (10 from women with GDM and 10 from women with non-GDM). A moderate correlation was observed between milk volume and postpartum time, with no significant differences between the GDM and non-GDM groups. Colostrum from women with GDM had lower protein content compared with milk from women with non-GDM (3.8 ± 0.4 vs. 5.2 ± 0.5 g/dL, *p* = 0.02) and transitional milk (1.4 ± 0.2 vs. 2.2 ± 0.2 g/dL, *p* = 0.02). Transitional milk from GDM group showed higher total fat (5.7 ± 1.8 vs. 2.0 ± 0.4 g/100 g, *p* = 0.05) and fat-to-protein ratio (3.9 ± 1.1 vs. 1.0 ± 0.3, *p* = 0.02), along with an increased ω-6/ω-3 ratio driven by higher linoleic acid and lower α-linolenic acid concentrations. **Conclusions**: GDM was associated with variations in breast milk protein and FA profiles with a potential negative impact on the newborn’s neurodevelopment.

## 1. Introduction

Gestational Diabetes Mellitus (GDM), defined as carbohydrate intolerance first recognized during pregnancy [1], is one of the most prevalent pregnancy-related complications, with a global prevalence of approximately 7% and rates reaching up to 17.7% in Mexico [2,3] This condition is associated with an increased risk of adverse outcomes such as cardiometabolic alterations for both the mother and fetus, in the short, medium, and long term.

Human milk composition is influenced by several maternal factors such as diet, maternal health, antidiabetic therapy and mammary gland physiology [4]. GDM involves an impaired energy metabolism that may affect human milk synthesis and secretion of key milk components [5]. From a physiological perspective, hormonal changes from mid-gestation onward reduce insulin sensitivity, a process normally compensated by increased insulin secretion [6]. In women with GDM, this compensatory mechanism is insufficient, leading to exacerbated insulin resistance. In addition, insulin resistance is further aggravated by elevated proinflammatory cytokines such as tumor necrosis factor-α (TNF-α) and interleukin-6 (IL-6), which may impair lipid transport to the mammary gland and disrupt milk composition [7,8]. Hormones involved in glucose regulation and lactation, including insulin, prolactin, cortisol and placental lactogen, also contribute to these metabolic alterations [9,10].

Beyond glycemic dysregulation, GDM induces broader metabolic alterations, including elevated triglycerides and specific diacylglycerols, consistent with increased de novo lipogenesis during pregnancy [11]. These disruptions in maternal lipid metabolism directly affect human milk composition, as the mammary gland captures and transfers to the neonate circulating fatty acids (FAs) derived from both dietary intake and endogenous synthesis. Insulin receptor expression in the mammary gland increases during lactation, and insulin plays a key role in the physiological regulation of lipogenesis; consequently, impaired insulin sensitivity alters the activity of critical enzymes involved in long-chain FA synthesis [12,13].

FAs play a crucial role in the structural and functional development of cellular membranes in the newborn [14,15]. Polyunsaturated fatty acids (PUFAs), particularly omega-3 (ω-3: α-linolenic acid [ALA], eicosapentaenoic acid [EPA], and docosahexaenoic acid [DHA]) and omega-6 (ω-6: linoleic acid [LA] and arachidonic acid [ARA]), are considered essential for optimal neurodevelopment [16]. Since breast milk represents the sole source of ω-3 and ω-6 PUFAs for neonates, ensuring adequate concentrations of these FAs is of utmost importance for infant neurodevelopment and metabolic programming. In women with GDM, however, the altered lipid metabolism inherent to this condition may compromise the FA profile of breast milk, potentially leading to suboptimal PUFA delivery to the newborn [17]. Despite this, the specific composition of breast milk in this population remains insufficiently characterized. A deeper understanding of its macronutrient and FA content is therefore essential, not only to identify nutritional gaps, but also to recognize critical windows of opportunity for targeted dietary counseling and supplementation strategies in mothers with GDM, ultimately supporting optimal infant growth and development.

The majority of research on milk composition in women with GDM has focused on mature milk, where higher fat and protein contents have been reported compared with milk from women without this condition, along with alterations in FA profiles [18,19]. Colostrum and transitional milk have been less frequently studied, primarily due to challenges in sample collection, high compositional variability, and limited sample volumes. These early lactation stages are especially significant because they exhibit the most substantial changes in milk composition [20,21].

Given the critical role of FAs supplied through breast milk during early life, and the limited and inconsistent evidence on milk composition in GDM, particularly regarding colostrum and transitional milk, this study aimed to address this knowledge gap by comparing FAs and protein concentrations in colostrum and transitional milk from women with GDM and those with uncomplicated pregnancies. Based on evidence linking GDM to altered lipid metabolism, including changes in ω-3 and ω-6 PUFA profiles [22], we hypothesized that colostrum and transitional breast milk from women with GDM would exhibit lower ω-3 PUFA concentrations and higher ω-6 PUFA concentrations compared with milk from women with non-GDM pregnancies.

## 2. Materials and Methods

### 2.1. Study Population

This was a prospective, observational, analytical, comparative, cross-sectional study conducted between January 2023 and December 2024 at the Maternal–Fetal Medicine Service at the Department of Obstetrics and Gynecology and the Child Wellness Unit of the Hospital General de México “Dr. Eduardo Liceaga” in Mexico City. Eligible participants included women aged 18 years or older with either non-GDM pregnancies or diagnosed GDM. The exclusion criteria included: (1) preeclampsia, (2) systemic diseases (including neurological, thyroid, rheumatological, renal, hepatic, and digestive conditions), (3) perinatal infections, (4) supplementation with ω-3 or ω-6 FA during pregnancy.

The study was approved by the Hospital’s Institutional Research, Ethics, and Biosafety for Human Research Committee (DI/23/303/05/24). Participants were recruited within the first 12–24 h after vaginal or cesarean delivery, with no obstetric or neonatal complications, while in rooming-in care. After being informed of the study objectives, mothers signed a written informed consent form. All participants were offered infant growth monitoring at our child clinic and invited to attend psychoeducational parenting workshops throughout the subsequent 18-month follow-up period.

### 2.2. GDM Diagnosis

After 24 weeks of gestation, GDM was identified using a 75-g Oral Glucose Tolerance Test (OGTT), with plasma glucose thresholds of ≥92 mg/dL in the fasting state, ≥180 mg/dL at 1 h, or ≥153 mg/dL at 2 h. GDM was defined when at least one of these thresholds was met or exceeded. All data were obtained from participants’ medical records.

### 2.3. Milk Sample Collection

Once written informed consent was obtained from the participating mothers, milk samples were collected using a hospital-grade electric breast pump (Medela Lactina^®,^ Medela AG, Baar, Switzerland) with sterile cups, connectors, and tubing. The breast selected for sampling was chosen according to the interval since the last feeding, ensuring that at least 1.5 h had elapsed. Prior to extraction, a standardized nipple and areola cleaning protocol was implemented.

Colostrum samples (collected between 0 and 5 days postpartum) were obtained using the automated 15-min program of the electric breast pump, which was selected to minimize mechanical stress on the nipple and ensure maternal comfort during the early postpartum period. For transitional milk (6–14 days), complete breast emptying was performed to obtain a homogeneous sample, as composition varies between foremilk and hindmilk. Milk volume and postpartum time were recorded and included in the statistical analysis. Milk was collected in sterile tubes, aliquoted into 1.5 mL sterile vials, and stored at −80 °C until analysis. Milk sample analyses were performed at the Instituto Nacional de Ciencias Médicas y Nutrición “Salvador Zubirán”, Mexico City.

### 2.4. Fatty Acids Analysis

Total lipids were extracted from milk samples using the Folch method [23]. Lipid extracts were subsequently derivatized to fatty acid methyl esters (FAMEs) using methanol, toluene, and 2% sulfuric acid, with decanoic acid as the internal standard. Samples were incubated at 90 °C for 2 h, followed by hexane extraction, and subsequently analyzed using a gas chromatograph equipped with a DB-225 column (PEAK Scientific, Inchinnan, UK). FA were identified by comparison with retention times of commercial standards and quantified relative to the internal standard [24].

### 2.5. Protein Analysis

Protein concentration was determined using the Bradford assay and calculated from the bovine serum albumin (BSA) standard curve and expressed in g/dL. To ensure measurements within the assay’s linear range, colostrum and transitional milk were analyzed at 1:76 and 1:15 dilutions, respectively [25].

### 2.6. Sociodemographic and Clinical Variables

The Hospital General de México primarily serves a vulnerable population with limited economic resources and no access to social security coverage. The women included in this study resided in urban surrounded areas and presented a highly homogeneous socioeconomic, cultural, and ethnic background.

Sociodemographic (maternal age) and clinical variables (pre-pregnancy weight, maternal height, parity, pharmacological treatment, mode of delivery, birth weight, and birth length) were obtained from clinical records. To characterize the study sample, maternal age was analyzed as a continuous variable and was also categorized according to the criteria of the International Federation of Gynecology and Obstetrics (FIGO), which defines advanced maternal age as pregnancy occurring at 35 years of age or older.

Treatment received was categorized into three groups: (1) dietary management, (2) metformin monotherapy, and (3) combined pharmacological therapy with metformin and insulin.

### 2.7. Statistical Analysis

Continuous variables were assessed for normality using the Shapiro–Wilk test, complemented by visual inspection of histograms and Q–Q plots. Normally distributed variables were expressed as the mean ± standard error (SE), whereas non-normally distributed variables were reported as median (minimum–maximum). Categorical variables were expressed as frequencies and proportions. Outliers were identified using the interquartile range method (IQR × 1.5) and were excluded only when exceeding this threshold, ensuring that less than 10% of the data were removed.

To account for the potential influence of postpartum time during early lactation, analysis of covariance (ANCOVA) was performed with milk volume as the dependent variable and postpartum time (hours) incorporated as a continuous linear covariate, thereby allowing estimation of its linear contribution while simultaneously testing fixed group effects. An overall model including all samples was first conducted with lactation stage (colostrum vs. transitional milk) as the fixed factor. Because milk volume differed markedly according to lactation stage, subsequent ANCOVA models were stratified by lactation stage; in these models, maternal group (non-GDM vs. GDM) was entered as the fixed factor, and postpartum time was retained as the continuous linear covariate. No additional covariates were incorporated, as these models were specifically designed to evaluate the influence of postpartum time together with group-related differences in milk volume.

To evaluate differences in milk protein, FA content, and other quantitative variables between the non-GDM and GDM groups, Student’s *t*-test was used for normally distributed variables, whereas the Mann–Whitney U test was used for non-normally distributed variables. Differences in categorical variables were evaluated using chi-square test.

In addition, we assessed whether milk composition differed within the GDM group according to the treatment received (dietary management, metformin monotherapy, or combined therapy with metformin and insulin) using a one-way ANOVA.

Statistical significance was set at *p* < 0.05. All statistical analyses were performed using IBM SPSS Statistics v29.0 (IBM Corp., Armonk, NY, USA) and SigmaPlot v14.0.

## 3. Results

### 3.1. Study Sample Characteristics

**Figure 1:**
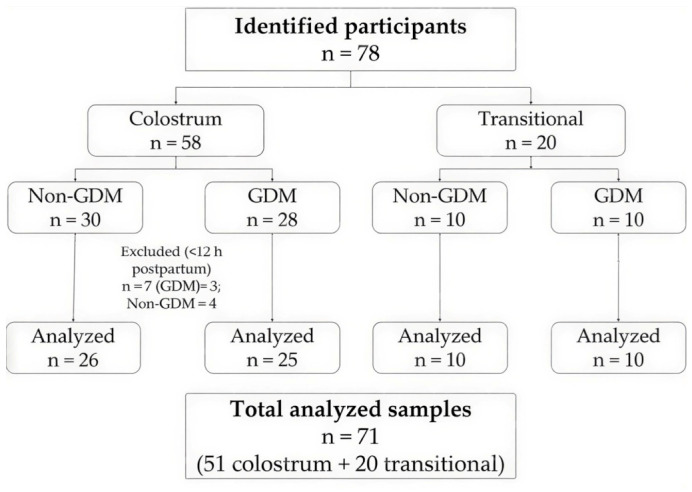
Distribution of study participants and analyzed milk samples.

A total of 78 participants were included in the study (Figure 1). Of the total sample, 58 women provided colostrum samples (30 non-GDM and 28 GDM), and 20 provided transitional milk samples (10 per pregnancy category). Seven colostrum samples were excluded from the analysis because they were collected within the first 12 h postpartum, which resulted in high measurement variability. Consequently, a total of 71 samples were included in the final analysis. Regarding women with GDM, 14.2% were managed with dietary intervention alone, 63.0% received metformin monotherapy, and 22.8% required combined pharmacological therapy consisting of metformin and insulin.

**Figure 2:**
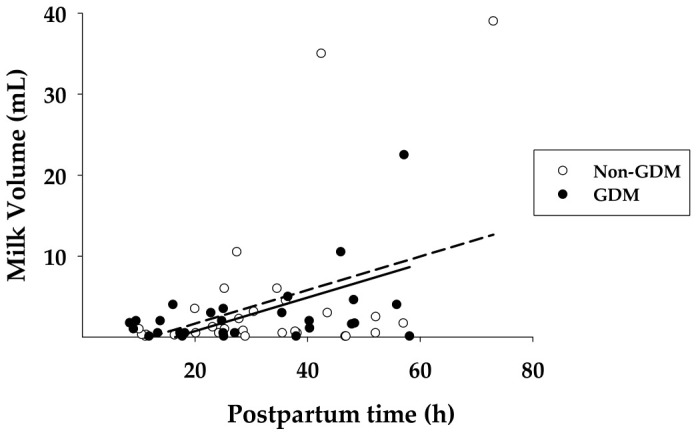
Association between postpartum time and milk volume in colostrum according to maternal group. Individual milk volume values are plotted across postpartum hours for women without gestational diabetes mellitus (Non-GDM, open circles) and with gestational diabetes mellitus (GDM, closed circles), with fitted adjusted linear trends from the ANCOVA model (dashed line, Non-GDM; continuous line, GDM). Postpartum time showed a significant linear effect (*p* < 0.001), whereas maternal group showed no significant effect (*p* = 0.592).

Given the marked variability in sample collection times during the early postpartum period, additional analyses were performed to determine whether postpartum time influenced milk volume. ANCOVA including all samples showed that adjusted milk volume was significantly higher in transitional milk than in colostrum (*p* = 0.009), whereas the linear effect of postpartum time was not significant in the overall model (*p* = 0.628). Therefore, subsequent analyses were stratified by lactation stage (Table S1). In colostrum, adjusted milk volume did not differ between non-GDM and GDM groups (4.0 ± 1.2 vs. 3.0 ± 1.3 mL, *p* = 0.592), but the linear effect of postpartum time was significant (*p* < 0.001; Figure 2). In transitional milk, adjusted milk volume was similar between non-GDM and GDM groups (24.0 ± 5.2 vs. 22.7 ± 5.2 mL, *p* = 0.865), and the linear effect of postpartum time was not significant (*p* = 0.842).

### 3.2. Maternal and Newborn Characteristics

Maternal demographic and anthropometric characteristics are presented in Table 1. Women with GDM were significantly older than those in the non-GDM group (median 31 vs. 25.5 years, *p* < 0.05). However, when participants were categorized as younger or older than 35 years, the difference between groups was only marginal. In addition, no significant differences were observed in the number of samples collected at each stage of lactation according to maternal age category and GDM status. No significant differences were observed in maternal height, pregestational weight, pre-pregnancy BMI, weight gain during pregnancy, or parity.

Newborn characteristics did not differ significantly between groups (Table 2). Median gestational age at delivery was 38 weeks in both groups. Birth weight and length were comparable between groups.

### 3.3. Colostrum Composition

**Figure 3:**
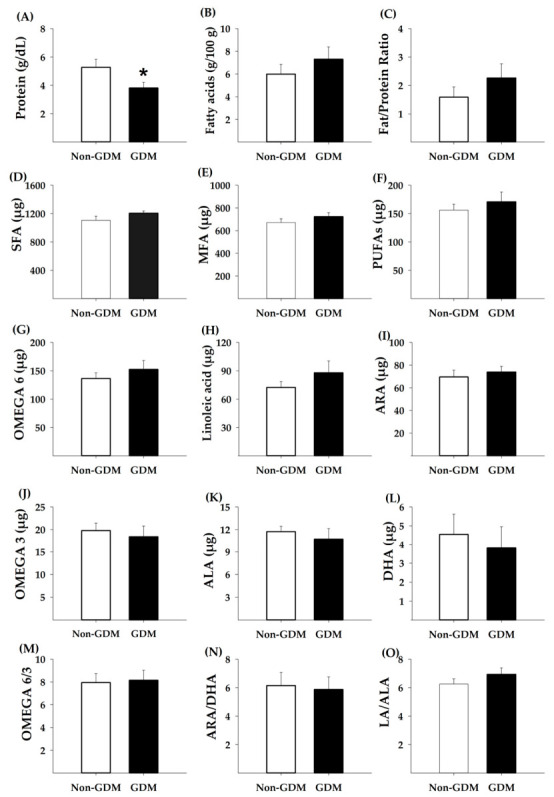
Macronutrient composition and fatty acid profiles of colostrum according to maternal GDM status. (**A**) Protein concentration, (**B**) total fat content, (**C**) fat-to-protein ratio, (**D**–**F**) major fatty acid classes (SFA, MUFA, PUFAs), (**G**–**I**) ω-6 fatty acids (total ω-6, linoleic acid, ARA), (**J**–**L**) ω-3 fatty acids (total ω-3, ALA, DHA), and (**M**–**O**) selected fatty acid ratios (ω-6/ω-3, ARA/DHA, LA/ALA). Data are presented as the mean ± SE. * *p* < 0.05 vs. non-GDM group.

Macronutrient and FA profiles of colostrum from women with non-GDM and GDM pregnancies are shown in Figure 3. Protein concentration was significantly lower in the GDM group compared with the non-GDM group (3.8 ± 0.4 vs. 5.2 ± 0.5 g/dL, *p* = 0.02). No differences were observed in FA concentrations.

When stratified by treatment modality, no significant differences in colostrum composition were identified among women with GDM.

### 3.4. Transitional Milk Composition

**Figure 4:**
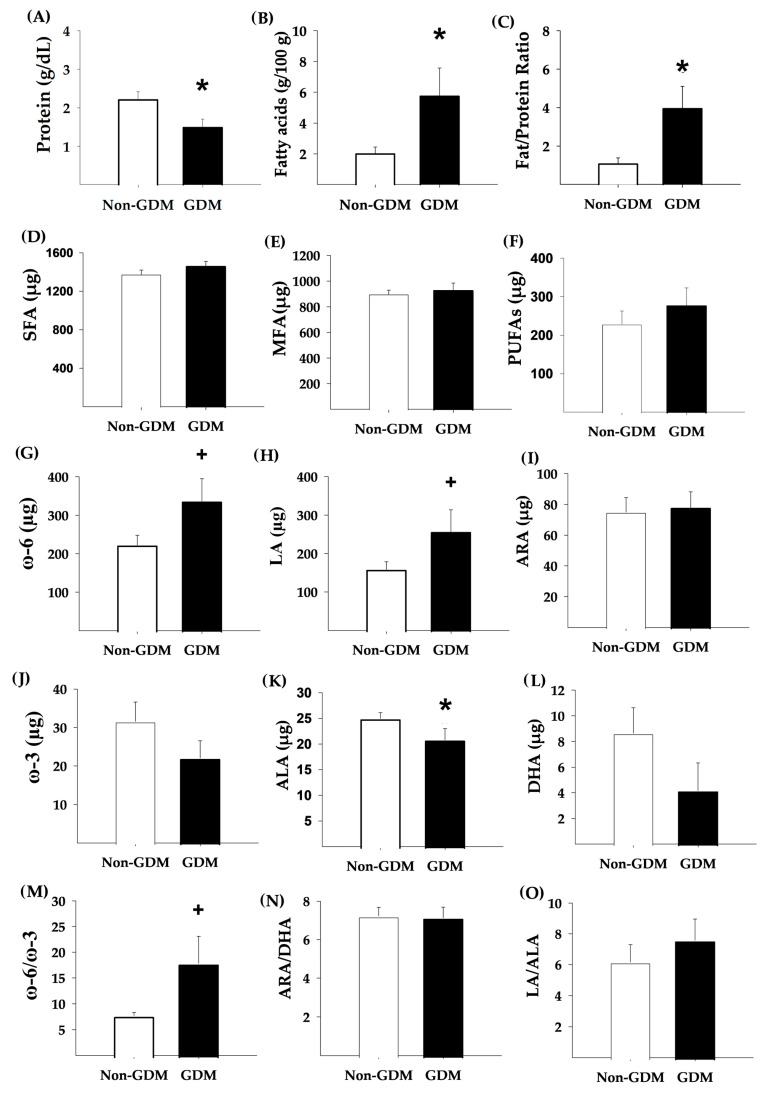
Macronutrient composition and fatty acid profiles of transitional milk according to maternal GDM status. (**A**) Protein concentration, (**B**) total fat content, (**C**) fat-to-protein ratio, (**D**) total SFA, (**E**) total MUFA, (**F**) total PUFAs, (**G**) total ω-6 fatty acids, (**H**) linoleic acid (LA), (**I**) arachidonic acid (ARA), (**J**) total ω-3 fatty acids, (**K**) α-linolenic acid (ALA), (**L**) docosahexaenoic acid (DHA), (**M**) ω-6/ω-3 ratio, (**N**) ARA/DHA ratio, and (**O**) LA/ALA ratio. Data are presented as the mean ± SE. * *p* < 0.05 vs. non-GDM group; + *p* = 0.06 indicates marginal significance.

Macronutrient composition and FA profiles of transitional milk from women with non-GDM and GDM pregnancies are shown in Figure 4. Protein concentration was significantly lower in the GDM group compared with the non-GDM group (1.4 ± 0.2 vs. 2.2 ± 0.2 g/dL, *p* = 0.02). In contrast, total fat content (5.7 ± 1.8 vs. 2.0 ± 0.4 g/100 g, *p* = 0.05) and the fat-to-protein ratio (3.9 ± 1.1 vs. 1.0 ± 0.3, *p* = 0.02) were significantly higher in women with GDM.

No significant between-group differences were observed in total SFA, MUFA, or PUFA concentrations. Transitional milk from women with GDM exhibited higher total ω-6 and lower ω-3 fatty acid concentrations, resulting in a higher ω-6/ω-3 ratio; however, this difference was marginally significant (17.8 ± 5.2 vs. 7.7 ± 0.6, *p* = 0.06).

Among individual FAs, linoleic acid concentrations marginally higher in the GDM group (257.3 ± 56.4 vs. 158.5 ± 20.0, *p* = 0.06), whereas α-linolenic acid (ALA) concentrations were significantly lower compared with the non-GDM group (19.3 ± 1.8 vs. 25.0 ± 1.1 µg, *p* = 0.02). No significant differences were observed in DHA or ARA concentrations between groups.

When stratified by treatment modality, no significant differences in transitional milk composition were identified among women with GDM.

## 4. Discussion

This study demonstrates that GDM influences human milk composition during the early stages of breastfeeding. Specifically, differences in protein content and FA profiles were observed between the GDM and non-GDM groups, mainly in transitional milk. Milk from women with GDM was characterized by lower protein concentrations and an imbalance in PUFAs, particularly reflected by an increased in ω-6/ω-3 ratio. These alterations could potentially predispose infants to limitations in growth [26] and neurodevelopment [27].

Breast milk is a dynamic fluid that continuously adapts its composition to meet the newborn’s needs. Milk composition is influenced by many factors, such as the mother’s age [28,29], ethnicity [30], nutrition and maternal health [31], method of delivery [32], time of day [33], lactation stage, and environmental factors [34]. It is for this reason that findings in the literature remain inconsistent, likely reflecting the considerable heterogeneity in sampling conditions and methodological approaches across studies.

Studies on breastfeeding among women with GDM are controversial. Some reports show no differences between women with GDM and those without; however, others indicate a shorter duration of breastfeeding, difficulties initiating or maintaining breastfeeding, or lower breastfeeding rates in women with GDM [35].

Furthermore, milk production is another variable that may fluctuate under different physiological conditions, thereby affecting milk volume and, consequently, the amount of macronutrients available to the newborn. Experimental studies in animal models suggest that obese females produce smaller volumes of more energy-dense milk, with a lower protein-to-fat ratio and an altered FA profile characterized by lower DHA and higher saturated and omega-6 fatty acids. As a result, offspring consume less milk overall and may receive lower amounts of protein and DHA, while being exposed to higher levels of total fat and pro-inflammatory fatty acids [36]. In humans, accurately monitoring and assessing maternal milk production and infant milk intake across the different stages of lactation remains challenging.

In the present study, GDM had no significant effect on milk volume, suggesting that although milk composition may be altered in this condition, milk production is not substantially compromised. This finding contrasts with previous reports describing delayed lactogenesis and reduced milk volume in women with GDM [37].

We identified that colostrum from women with GDM had lower protein content. In contrast to this finding, a recent meta-analysis [18], estimated a slightly higher protein content in colostrum from mothers with GDM compared to non-GDM mothers. However, consistent with our results, no differences were found in total lipid content. Other studies conducted in Poland and Sweden have not found differences in either protein or total fat content in colostrum [38]. In contrast to findings from a study conducted in Israel [39], which reported higher concentrations of four ω-6 PUFAs in colostrum from women with GDM, we did not observe significant differences in any FA.

Regarding transitional milk, lower protein concentrations were also observed in the GDM group. However, the limited evidence on the impact of GDM on transitional milk composition remains inconsistent. A study conducted in Athens similarly reported lower protein concentrations in women with GDM [21], whereas another study from Israel did not observe differences in this macronutrient [40]. Evidence from animal models have found that insulin signaling plays a role in the regulation of milk protein synthesis [41]; therefore, insulin resistance in GDM may alter these pathways and potentially contribute to changes in milk protein content.

Moreover, transitional milk in the GDM group exhibited greater total fat concentrations and an elevated ω-6/ω-3 ratio, driven by marginally higher ω-6 FA levels (*p* = 0.06). Prior research has shown that lipidomic signatures associated with GDM can persist up to a year postpartum. Such prolonged disturbances in lipid metabolism could sustainably alter the FA composition of breast milk from early to later stages of lactation [42].

FA content in human milk mainly depends on three sources: (1) de novo synthesis in the mammary gland, (2) FAs derived from maternal body stores, and (3) maternal dietary intake [20,43]. As previously mentioned, metabolic and hormonal alterations in GDM may affect both de novo FA synthesis in the mammary gland and the contribution of FAs derived from maternal body stores, for example through increased lipolysis. Beyond these mechanisms, it is important to assess whether women with GDM exhibit specific dietary patterns that may further contribute to FA profile in human milk.

Previous studies evaluating the effect of maternal age on FA concentrations in colostrum and transitional milk have reported inconsistent findings. Evidence suggests that maternal age may influence FA composition during early lactation, though the specific lactation stage affected remains inconsistent across reports [28,29]. Because women with GDM in our study were older on average than women without GDM, maternal age may have partially contributed to the higher FA concentrations observed in the GDM group, particularly in transitional milk. These findings are consistent with previous reports suggesting that increasing maternal age is associated with higher milk FA concentrations during the early stages of lactation.

A study of mature breast milk found that FA concentrations varied across Asian ethnic groups despite similar maternal omega-3 and omega-6 intake, suggesting that ethnicity, beyond diet, may influence FA composition [30]. Therefore, the differences observed between the present study and previous reports may be partially explained by ethnic differences.

Management of GDM includes lifestyle and behavioral interventions as well as pharmacological therapies such as insulin or metformin [44]. Evidence regarding the effect of pharmacological treatments on breast milk composition remains limited. Interestingly, we did not observe differences in milk macronutrient content in colostrum or transitional milk from mothers with GDM according to the treatment received. A recent study reported that transitional milk from insulin-treated mothers exhibited a distinct metabolomic profile, characterized by alterations in glycolytic intermediates, purine metabolism, and oxidative pathways [45]. While these metabolic alterations may reflect differences in mammary gland metabolism and could be associated with changes in milk macronutrient composition, evidence in this area remains limited.

The composition of colostrum and transitional milk is poorly characterized and inconsistent across studies. Colostrum sample collection poses particular challenges, owing to the small volumes available and the high inter-individual variability in its composition. Additionally, colostrum has been largely excluded from research, as its biochemical composition changes rapidly during the early postpartum period. Consistent with this observation, colostrum samples collected within the first 12 h postpartum were excluded from our analysis due to their high variability, which limited reliable interpretation. Several parameters showed numerical differences that did not reach statistical significance, likely attributable to the rapid physiological changes characteristic of early lactation. Notably, these numerical trends became statistically significant in transitional milk, suggesting that the magnitude of change consolidates over time [4,15].

With respect to transitional milk, although sample collection is generally more feasible, to our knowledge there are no studies that have specifically examined fat content and FA composition in transitional milk from women with GDM. Nevertheless, further studies with larger sample sizes and in diverse populations are needed to better characterize GDM related alterations in milk composition. In addition, longitudinal studies are required to evaluate the impact of these imbalances in protein and FA concentrations on child growth and development.

This study has some limitations that should be considered. First, the sample was non-probabilistic and derived from a single-center study conducted in a specific population, which may limit the generalizability of the findings. Second, maternal dietary intake, physical activity, use of dietary supplements as well as postpartum metabolic markers, were not assessed, limiting our ability to evaluate their potential influence on milk composition. Finally, given the relatively small number of transitional milk samples, findings in this subgroup, especially those with marginal significance, should be regarded as preliminary and warrant replication in larger cohorts. Nevertheless, the inclusion of both colostrum and transitional milk provides valuable insight, as these lactation stages remain understudied.

## 5. Conclusions

Protein composition in colostrum and transitional milk differed between women with and without GDM. Specifically, colostrum from women with GDM exhibited lower protein content, whereas transitional milk from women with GDM showed a higher fat-to-protein ratio.

An imbalance in FA concentrations in breast milk from mothers with GDM could potentially affect offspring neurodevelopment, with possible implications for memory, learning, anxiety, and behavior. These findings underscore the need for future longitudinal studies to evaluate the potential long-term implications for offspring health. Furthermore, our results highlight the importance of developing targeted nutritional and metabolic intervention strategies during pregnancy and lactation to optimize milk composition and support favorable infant growth and neurodevelopment.

## Figures and Tables

**Table 1:** Maternal characteristics of the analyzed study population according to GDM status.

	Non-GDM	GDM	*p*-Value
	(n = 36)	(n = 35)	
Age (years)	25.5 (18–39)	31 (18–45)	0.01 *
**Maternal age category**	**n (%)**	**n (%)**	0.06
Non advanced age	33 (91.7)	25 (71.4)	
Advanced age	3 (8.3)	10 (28.6)	
**Milk samples**	**n (%)**	**n (%)**	0.18
Colostrum, non-advanced age	24 (66.7)	18 (51.4)	
Colostrum, advanced age	2 (5.5)	7 (20)	
Transitional, non-advanced age	9 (25)	7 (20)	
Transitional, advanced age	1 (2.8)	3 (8.6)	
Height (m)	1.56 (1.38–1.72)	1.57 (1.43–1.73)	0.56
Pre-pregnancy weight (kg)	62.5 (38–116)	68 (40–91)	0.78
Weight gain during pregnancy (kg)	9.5 (0–25)	6.5 (2–21)	0.73
**Pre-pregnancy BMI categories**	**n (%)**	**n (%)**	0.33
Normal weight	17 (47.2)	12 (34.3)	
Overweight	7 (19.4)	12 (34.3)	
Obesity	12 (33.3)	11 (31.4)	
**Parity**	**n (%)**	**n (%)**	0.74
Primiparous	13 (36.1)	15 (42.9)	
Multiparous	23 (63.9)	20 (57.1)	

Data are presented as median (minimum–maximum) for quantitative variables and as frequencies with percentages for categorical variables. For maternal characteristics, “n” corresponds to the number of mothers in each group. For milk sample categories, “n” corresponds to the number of samples stratified by lactation stage and maternal age category within each group. Non advanced age (˂35 years), Advanced age (≥35 years). Non-GDM: non-gestational diabetes; GDM: gestational diabetes mellitus. * *p* < 0.05.

**Table 2:** Newborn characteristics according to maternal GDM status.

	Non-GDM (n = 36)	GDM (n = 35)	*p*-Value
Birth weight (kg)	2.8 (1.7–4.2)	2.9 (1.5–4.5)	0.57
Birth length (cm)	49 (41–58)	49 (42–52)	0.39
Gestational age (weeks)	38.5 (32–42.4)	38 (36–41.6)	0.13
**Mode of delivery**	**n (%)**	**n (%)**	0.28
Vaginal delivery	17 (47.2)	12 (34.2)	
Cesarean section	19 (52.5)	23 (65.7)	

Data are presented as medians (minimum-maximum) for quantitative variables and as frequencies and percentages for categorical variables. Non-GDM: non-gestational diabetes; GDM: gestational diabetes mellitus.

## Data Availability

The original contributions presented in this study are included in the article/Supplementary Material. Further inquiries can be directed to the corresponding authors.

## Supplementary Materials

The following supporting information can be downloaded at: https://www.mdpi.com/article/10.3390/nu18111803/s1, Table S1: Summary of ANCOVA models assessing the linear effect of postpartum time on milk volume according to lactation stage and maternal GDM status.

## Abbreviations

The following abbreviations are used in this manuscript:

GDM	Gestational diabetes mellitus
FA	Fatty acids
PUFAs	Polyunsaturated fatty acids
SFA	Saturated fatty acids
MUFA	Monounsaturated fatty acids
ALA	α-linolenic acid
EPA	Eicosapentaenoic acid
DHA	Docosahexaenoic acid
LA	Linoleic acid
ARA	Arachidonic acid
OGTT	Oral glucose tolerance test
ANCOVA	Analysis of covariance
TNF-α	Tumor necrosis factor alpha
IL-6	Interleukin 6
FAMEs	Fatty acid methyl esters
SE	Standard error
