# Supplementary material for: Omega Fatty Acid and Protein Profiles of Colostrum and Transitional Milk in Mexican Women With and Without Gestational Diabetes: A Cross-Sectional Study
Source: Nutrients. 2026 Jun 3;18(11):1803. doi: 10.3390/nu18111803 (PMC13258512; doi:10.3390/nu18111803)
Supplement: Supplementary file 1 [file nutrients-18-01803-s001.zip › nutrients-4291990-supplementary.pdf]

Table S1. Summary of ANCOVA models assessing the linear effect of postpartum time on milk volume according to lactation stage and maternal GDM status.

Model	Fixed factor	Covariate	Adjusted means (mL)	Fixed factor effect	Covariate effect
All milk samples	Lactation stage	Postpartum time in hours	Colostrum: 4.2 ± 2.0<br>Transitional milk: 21.2 ± 4.9	$F_{(1,75)}$ = 7.11<br>p = 0.009	$F_{(1,75)}$ = 0.24<br>p = 0.628
Colostrum only	Maternal group	Postpartum time in hours	Non-GDM: 4.0 ± 1.2<br>GDM: 3.0 ± 1.3	$F_{(1,55)}$ = 0.29<br>p =0.592	$F_{(1,55)}$ = 12.95<br>p < 0.001
Transitional milk only	Maternal group	Postpartum time in ours	Non-GDM: 24.0 ± 5.2<br>GDM: 22.7 ± 5.2	$F_{(1,17)}$ = 0.03<br>p = 0.865	$F_{(1,17)}$ = 0.04<br>p = 0.842

Data are presented as adjusted means ± standard error. ANCOVA, analysis of covariance; GDM, gestational diabetes mellitus.
